# Readmissions and Emergency Room Visits Following Outpatient and Inpatient Herniorrhaphies

**DOI:** 10.7759/cureus.96334

**Published:** 2025-11-07

**Authors:** Abirami Muthumani, Angel Rosario, Artem Shmelev, Yuri W Novitsky

**Affiliations:** 1 Surgery, Columbia University Vagelos College of Physicians and Surgeons, New York, USA; 2 Surgery, Stony Brook University, Stony Brook, USA

**Keywords:** complications, emergency room, hernia repair, readmissions, socioeconomic disparities

## Abstract

Background: Hospital readmissions following abdominal wall hernia repairs (HR) are recognized sources of additional morbidity and costs. Large-scale studies focus primarily on readmissions and do not capture emergency department visits (EDV), time-wise stratification of post-operative encounters, or outpatient HR. We aimed to determine the incidence, timing, and primary reasons for EDVs and readmissions by linking state-level databases.

Methods: Patients who underwent HR in Maryland in 2016-2017 were identified in linked state-level databases, covering 95% of all HR. Encounters were grouped by postoperative timing and admitting diagnoses. Predictors of postoperative encounters were determined.

Results: Of 26,215 patients undergoing HR (87.5% outpatient, 48.7% inguinal), 5,802 (22.1%) had at least one postoperative encounter (4,186 EDV, 1,415 readmissions). EDV comprised 81.0% (419) of encounters within the first 48 hours. Top reasons for EDV were urinary disorders (24.1%, 10.6% and 4.0% on postoperative days (PODs) 0-2, 3-7, and 8-30, respectively), pain (18.1%, 24.9%, 14.4%), and GI complaints (10.5%, 9.9%, 3.4%). Readmissions mainly occurred for GI complaints (15.3%, 19.9%, 6.9%), local surgical site infection (SSI) (5.1%, 15.5%, 26.8%), and respiratory complications (8.2%, 6.6%, 4.1%). The most significant predictors of postoperative encounters were non-private insurance and African American origin.

Discussion: ED visits and readmissions after herniorrhaphy remain major healthcare utilizations, mainly due to urinary disorders, pain, GI issues, and SSI. Insurance and race are linked to such encounters, indicating potential for targeted interventions.

## Introduction

Despite recent advances in abdominal wall reconstruction, postoperative readmissions remain a substantial source of morbidity and additional costs. For example, the reported nationwide readmission rate following ventral hernia repairs (HR) is 20% [1], which is higher than after complex oncologic procedures [2]. As a result, strategies to prevent readmissions after herniorrhaphies are exponentially drawing interest.

Most published-to-date studies of readmissions following ventral and inguinal HR utilize nationwide data, such as the National Readmission Database [3,4], other national registries [5-7], American College of Surgeons National Surgical Quality Improvement Program (ACS NSQIP) [8], or institutional data [9-11]. While these studies are useful for understanding inpatient HR, which presumably have higher complexity, they largely omit outpatient repairs and emergency department visits (EDVs), with rare exceptions [12]. Additionally, no studies describe a time-wise breakdown of postoperative encounters.

We aimed to fill the gaps in the current understanding of the type (readmission or EDV) and timing of unplanned postoperative encounters after HR. To achieve comprehensive coverage for both inpatient and ambulatory HR, as well as postoperative readmissions and EDVs, we linked several state-level administrative databases of the Healthcare Cost and Utilization Project (HCUP). We aimed to determine the incidence, timing, and primary reasons for EDVs and readmissions after HR. To our knowledge, integrating multiple HCUP databases for a comprehensive review of postoperative events represents a novel approach.

## Materials and methods

Data source

This was a retrospective cohort analysis evaluating HR (inpatient and outpatient), postoperative encounters (EDV vs readmission), and time to these encounters using the State Inpatient Database (SID), State Emergency Department Database (SEDD), and State Ambulatory Surgery and Services Databases (SASD) of Maryland, United States, 2016-2017, provided by the Agency for Healthcare Research and Quality, United States [13]. Institutional review board approval was waived as not required for the analysis of de-identified administrative data according to our institution’s policy.

At the time of statistical analysis, our institution did not have access to more updated versions of these databases. State-level databases cover up to 97% of all discharges within a state [14]. These databases contain revisit variables, including a de-identified unique patient number, which allows linking of encounters of different types (hospitalization, EDV, and outpatient surgery, excluding outpatient offices and urgent care centers), and a timing variable, enclosing time gaps between encounters. As such, one can reconstruct a timeline of most healthcare encounters of a single individual within one state within a given calendar year.

Study population

We selected all encounters with procedural codes for abdominal wall hernia repairs (International Classification of Diseases, 10th Revision, Procedure Coding System (ICD-10-PCS) for inpatient (SID) procedures and Current Procedural Terminology (CPT®) for outpatient (SASD) procedures (see Appendices). We included only patients with primary diagnoses of abdominal wall hernias in order to isolate initial hernia patients from those who underwent herniorrhaphy in combination with a different intervention, such as primary (non-mesh) repair of umbilical hernia during laparoscopic cholecystectomy. Encounters of patients who underwent HR were supplemented by their hospitalizations (SID) and EDVs (SEDD) by linking all encounters among all state-level databases as described above. We identified and removed all duplicate records. The final study database encompassed encounters with herniorrhaphies (referred to as “index encounters”) and all subsequent patients’ encounters (hospitalizations and EDVs) that occurred during the same calendar year.

Statistical considerations

All analyses were performed in IBM SPSS Statistics for Windows, version 22 (Released 2013; IBM Corp., Armonk, New York, United States) and R version 4.2.2 (2022; R Foundation for Statistical Computing, Vienna, Austria, https://www.R-project.org/). We computed time intervals between the index (operative) and first subsequent encounters using the SPSS LAG function. Timing of the first postoperative encounter was classified as follows: ≤2 days, 3-7 days, 8-30 days, 31-90 days, and >90 days. We determined the structure of primary diagnoses of the first postoperative encounters by timing and encounter type (EDV versus readmission). Additionally, we further analyzed the workup and therapy provided by EDs to the patients of the three most common diagnostic groups. Patients’ demographics and comorbidities, hernia type, surgical approach, and facility characteristics were compared between patients who had any 30-day postoperative encounter and those who did not. We utilized conventional statistical tools such as the Chi-squared and Mann-Whitney U tests.

We built two logistic regression models predicting 30-day postoperative readmission or EDVs. All potential predictors were critically assessed according to fundamental knowledge and clinical sense. Variables for the first model were selected by LASSO (least absolute shrinkage and selection operator) regression. This approach facilitates the selection of the most significant predictors and thus achieves a good balance of accuracy and simplicity (library “glmnet” within R) [15]. Variables for the second model were determined by backstep elimination based on likelihood ratios. For validation, we randomly split the input data into 80% training and 20% validation subsets and applied to the models 10-fold cross-validation (library “caret”).

## Results

**Figure 1:**
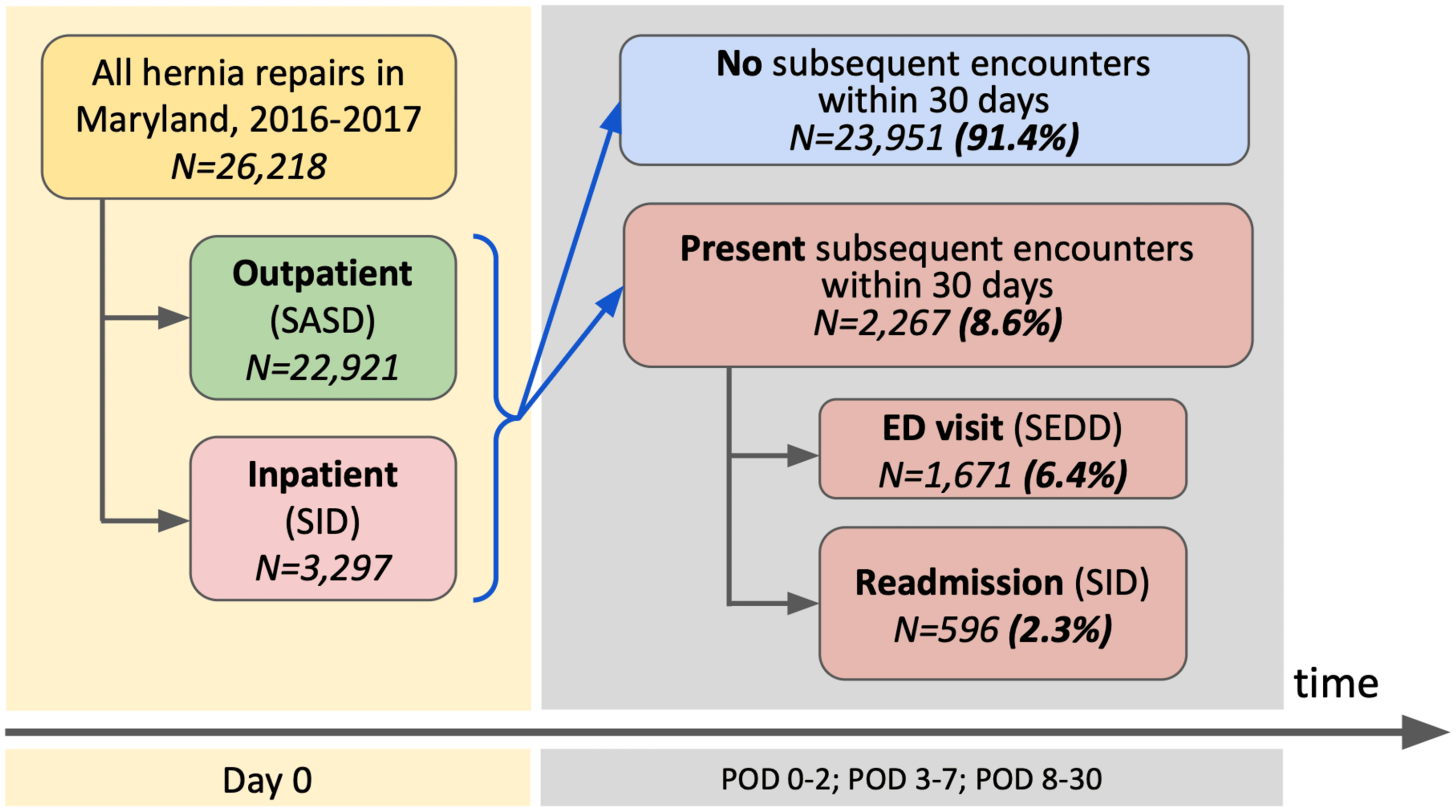
Study population design SASD: State Ambulatory Surgery and Services Databases; SID: State Inpatient Database; SEDD: State Emergency Department Database; POD: postoperative day

A total of 26,218 unique patients who underwent inpatient (n=3,297; 12.6%) or outpatient (n=22,921; 87.4%) abdominal wall HR (AHR) in Maryland during 2016 (n=12,749) and 2017 (n=13,469) were identified (Figure 1). Of those, 2,267 (8.6%) patients had a readmission or an EDV within 30 days after their hernia surgery. The timing of the first postoperative encounter by its type is listed in Table 1. The majority (81.0%) of postoperative encounters within the first 48 hours after surgery were EDVs. The fraction of EDVs within later encounters was in the 70.5-76.4% range. Within 90 days postoperatively, 953 (3.6%) patients were readmitted, and 2,733 (10.4%) visited the ED.

**Table 1:** Timing and type of the first postoperative encounter * fraction of a total number of encounters within a given time frame postoperatively

Timing of the 1^st^ post-op encounter	Type of encounter		
	Readmission	ED visit	Total
≤2 days	98 (19.0%*)	419 (81.0%)	517
3-7 days	181 (29.5%)	433 (70.5%)	614
8-30 days	317 (27.9%)	819 (72.1%)	1,136
31-90 days	357 (25.8%)	1,026 (74.2%)	1,383
>90 days	457 (23.6%)	1,479 (76.4%)	1,936
0-30 days	596 (26.3%)	1,671 (73.7%)	2,267
0-90 days	953 (25.9%)	2,733 (74.1%)	3,686

Individuals who had no 30-day ED or hospital encounters tended to be Caucasian males, with fewer comorbidities, privately insured, with higher income, and married. They had more groin HR than ventral HR, and no concomitant procedures. Conversely, those who had 30-day postoperative encounters were more likely to be African American patients, have Medicaid/Medicare, and have co-morbidities. They had more concomitant procedures, such as adhesiolysis, bowel resection, or cholecystectomy, and were more likely to receive blood products or ICU care. Expectedly, the incidence of the most common chronic conditions was higher in patients with higher readmissions or EDV. There was no difference in patients’ age or approach in bivariate analysis (Table 2).

**Table 2:** Bivariate comparisons of patients with and without 30-day postoperative encounters (ED visits or readmissions) ^a^ by independent samples Mann-Whitney U test (for skewed distributions) ^b^ by Chi-squared test ^c^ column percentages (i.e., 69% of patients without 30-day encounters were males) ^d ^Pearson Chi-Square statistic ^e ^Mann-Whitney U standardized test statistic (Z-score) SD: standard deviation; IQR: interquartile range; MIS: minimally-invasive surgery (laparoscopy/robotics); LOA: lysis of adhesions; OSA: obstructive sleep apnea; TIA/CVA: transient ischemic attack/cerebrovascular accident; BMI: body mass index; EtOH: ethanol (alcohol); ICU: intensive care procedures (e.g., mechanical ventilation, placement of central lines, administration of vasopressors, tube feeds); OSA: obstructive sleep apnea; CAD: coronary artery disease; HF: heart failure; MIS: minimally invasive surgery Please note that due to negligible fraction of missing data, total values within the variable may slightly differ.

Characteristic	No 30-day encounters (n=23,951)	Present 30-day encounters (n=2,267)	Test statistic	p-value
Age (years)			1.75^ e^	0.080 ^a^
Mean ± SD	53.5 ± 19.3	54.4 ± 20		
Median (IQR)	56 (43 – 67)	56 (43 – 69)		
Male sex	16,518 (69%)^c^	1,324 (58.4%)	106.2 ^d^	<0.001^ b^
Race/Ethnicity, n (%)			106.5 ^d^	<0.001^ b^
Caucasian	16,707 (70.2%)	1,380 (61.3%)		
African-American	5,229 (22%)	708 (31.4%)		
Hispanic	979 (4.1%)	93 (4.1%)		
Asian and Other	893 (3.8%)	71 (3.2%)		
Charlson comorbidity score, mean ± SD	0.51 ± 1.04	0.94 ± 1.47	16.3^ e^	<0.001^a^
Insurance, n (%)			388.5 ^d^	<0.001^ b^
Private	12,589 (52.6%)	772 (34.1%)		
Medicare	6,797 (28.4%)	843 (37.2%)		
Medicaid	3,318 (13.9%)	573 (25.3%)		
Other/Self-pay	1,247 (5.2%)	79 (3.5%)		
Single (vs. has partner), n (%)	10,246 (43.3%)	1,167 (52.1%)	64.9 ^d^	<0.001^ b^
Income quartile, n (%)			84.7 ^d^	<0.001^ b^
1st (lowest)	2,083 (8.8%)	304 (13.5%)		
2nd	2,482 (10.4%)	292 (13%)		
3rd	6,887 (29%)	656 (29.1%)		
4th (highest)	12,309 (51.8%)	999 (44.4%)		
Patient location, n (%)			41.7^ e^	<0.001^ b^
Metro area	2,908 (12.2%)	366 (16.2%)		
Large counties	16,662 (69.6%)	1,441 (63.6%)		
Smaller counties	4,359 (18.2%)	457 (20.2%)		
Hernia type, n (%)			90.3^ e^	<0.001^ b^
Isolated groin	11,106 (47.1%)	828 (37.4%)		
Isolated ventral/umbilical	11,525 (48.8%)	1,295 (58.4%)		
Groin + ventral/umbilical	970 (4.1%)	93 (4.2%)		
Total charges			21.0^ e^	<0.001^a^
Mean ± SD	8,388.6 ± 9,343.3	13,954.8 ± 20,292.5		
Median (IQR)	6,135 (4,333 – 9,328)	8,378 (5,346 – 14,407)		
Length of stay, mean ± SD	0.6 ± 2.2	1.9 ± 4.4	25.3^ e^	<0.001^a^
In-hospital mortality, n (%)	19 (0.1%)	0 (0%)	1.80 ^d^	0.179^ b^
Perioperative factors, n (%)				
Elective surgery	20,878 (91.4%)	1,770 (81.6%)	223.0 ^d^	<0.001^ b^
Outpatient surgery	21,309 (89%)	1,612 (71.1%)	601.0 ^d^	<0.001^ b^
MIS approach	7,734 (32.4%)	730 (32.3%)	0.016 ^d^	0.901^ b^
Intraoperative LOA	506 (2.1%)	168 (7.4%)	232.0 ^d^	<0.001^ b^
Concomitant GI‎/biliary procedure	254 (1.1%)	103 (4.5%)	187.0 ^d^	<0.001^ b^
Blood products administration	121 (0.5%)	58 (2.6%)	128.7 ^d^	<0.001^ b^
ICU care	337 (1.4%)	142 (6.3%)	272.3 ^d^	<0.001^ b^
Comorbidities, n (%)				
Cardiac (CAD, HF, arrhythmias)	3155 (13.2%)	503 (22.2%)	140.2 ^d^	<0.001^ b^
CVA/TIA	735 (3.1%)	154 (6.8%)	87.7 ^d^	<0.001^ b^
Chronic respiratory conditions and OSA	3712 (15.5%)	540 (23.8%)	105.6 ^d^	<0.001^ b^
Chronic renal disease	796 (3.3%)	162 (7.1%)	85.9 ^d^	<0.001^ b^
Diabetes mellitus	3015 (12.6%)	441 (19.5%)	85.3 ^d^	<0.001^ b^
Obesity (BMI>30)	2934 (12.3%)	462 (20.4%)	121.3 ^d^	<0.001^ b^
Dementia	159 (0.7%)	37 (1.6%)	26.2 ^d^	<0.001^ b^
Peptic ulcer disease	60 (0.3%)	12 (0.5%)	5.87 ^d^	0.015^ b^
Malignancy (any)	310 (1.3%)	49 (2.2%)	11.5 ^d^	0.001^ b^
Chronic liver disease	497 (2.1%)	95 (4.2%)	41.9 ^d^	<0.001^ b^
Cytopenia (any)	1080 (4.5%)	258 (11.4%)	201.9 ^d^	<0.001^ b^
Psychiatric disorders	2369 (9.9%)	408 (18%)	143.7 ^d^	<0.001^ b^
Chronic pain/opioids use	819 (3.4%)	154 (6.8%)	65.9 ^d^	<0.001^ b^
Tobacco, EtOH or drugs use	2004 (8.4%)	289 (12.7%)	49.8 ^d^	<0.001^b^
Rheumatologic disorders	244 (1%)	32 (1.4%)	3.06 ^d^	0.080^ b^
HIV/AIDS status	34 (0.1%)	5 (0.2%)	0.86 ^d^	0.353^ b^

Structure of 30-day postoperative encounters and associated charges

**Figure 2:**
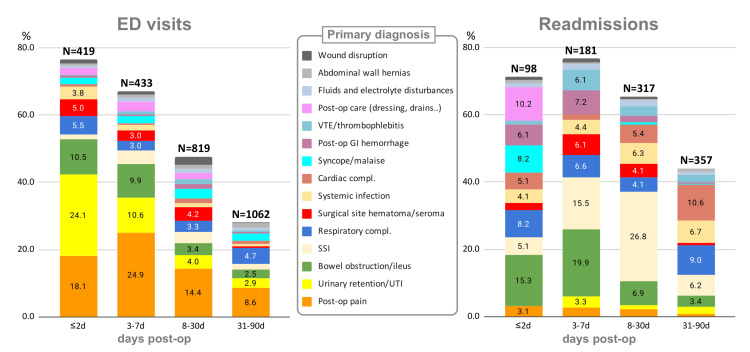
Structure of postoperative primary diagnoses with breakdown by timing of encounter “Bowel obstruction/ileus” group encompasses diagnoses of bowel obstruction (88/39.5%), constipation (84/37.7%), nausea and vomiting (35/15.7%), and ileus (12/5.4%). Urinary issues include urinary retention (106/46.5%) and UTI (60/26.3%). UTI: urinary tract infection; VTE: venous thromboembolism; SSI: surgical site infection

Primary diagnoses of EDVs or hospitalizations of patients are listed in Figure 2. Most frequent reasons for postoperative EDV were urinary complaints (n=101 (24.1%), n=46 (10.6%), and n=33 (4.0%) on postoperative days (PODs) 0-2, 3-7, and 8-30, respectively), followed by pain control issues (n=75 (18.1%), n=108 (24.9%), n=118 (14.4%), respectively), and delayed return of bowel function (n=44 (10.5%), n=43 (9.9%), n=28 (3.4%), respectively). Average charges for such EDVs were $958.6±1,316.0, $1,204.0±944.8, and $1,227.9±1,031.3, respectively. The fraction of these three most common conditions accounted for more than 50% of all EDVs within two days after surgery. This fraction progressively declined with later presentation times. Hospitalizations had a more diverse structure of admission diagnoses. Nonetheless, the top five admitting diagnostic groups (within 30-days postoperative) constituted 51.5% of all admissions: surgical site infection (n=118; 19.8%), delayed return of bowel function (n=73; 12.2%), respiratory complications (n=33; 5.5%), sepsis (n=32; 5.4%), surgical site hematoma or seroma (n=26; 4.4%), and cardiac complications (n=25; 4.2%). Charges for SSIs, GI complaints, and respiratory complications were $17,252±22,546, $15,404±23,556, and $12,362±13,38, respectively. A separate cost analysis was not performed due to the scope of this work. Diagnostic and therapeutic interventions provided to patients in the ED are summarized in Table 3.

**Table 3:** Diagnostic and therapeutic interventions provided in the ED during first visit within 30 days after herniorrhaphy ^a^ of all ED visits within 30 days postoperative

Interventions	Reason for ED visit		
	Post-operative pain (n=302, 18%^a^)	Urinary retention/UTI (n=180, 11%)	Delayed return of bowel function (n=115, 7%)
Analgesics	120 (39.7%)	25 (13.9%)	30 (26.1%)
Antiemetics	97 (32.1%)	17 (9.4%)	29 (25.2%)
Antibiotics	10 (3.3%)	20 (11.1%)	7 (6.1%)
IV fluids	35 (11.6%)	16 (8.9%)	17 (14.8%)
Diagnostic imaging	218 (72.2%)	56 (31.1%)	92 (80.0%)
Blood tests	254 (84.1%)	88 (48.9%)	80 (69.6%)
Urinalysis, urine culture	159 (52.6%)	144 (80.0%)	47 (40.9%)
Bladder catheterization	1 (0.3%)	13 (7.2%)	1 (0.9%)
Total charges, median (range)	1,008 (738 – 1,360)	697 (416 – 987)	897 (547 – 1,478)

Prediction of 30-day readmission or ED utilization

**Figure 3:**
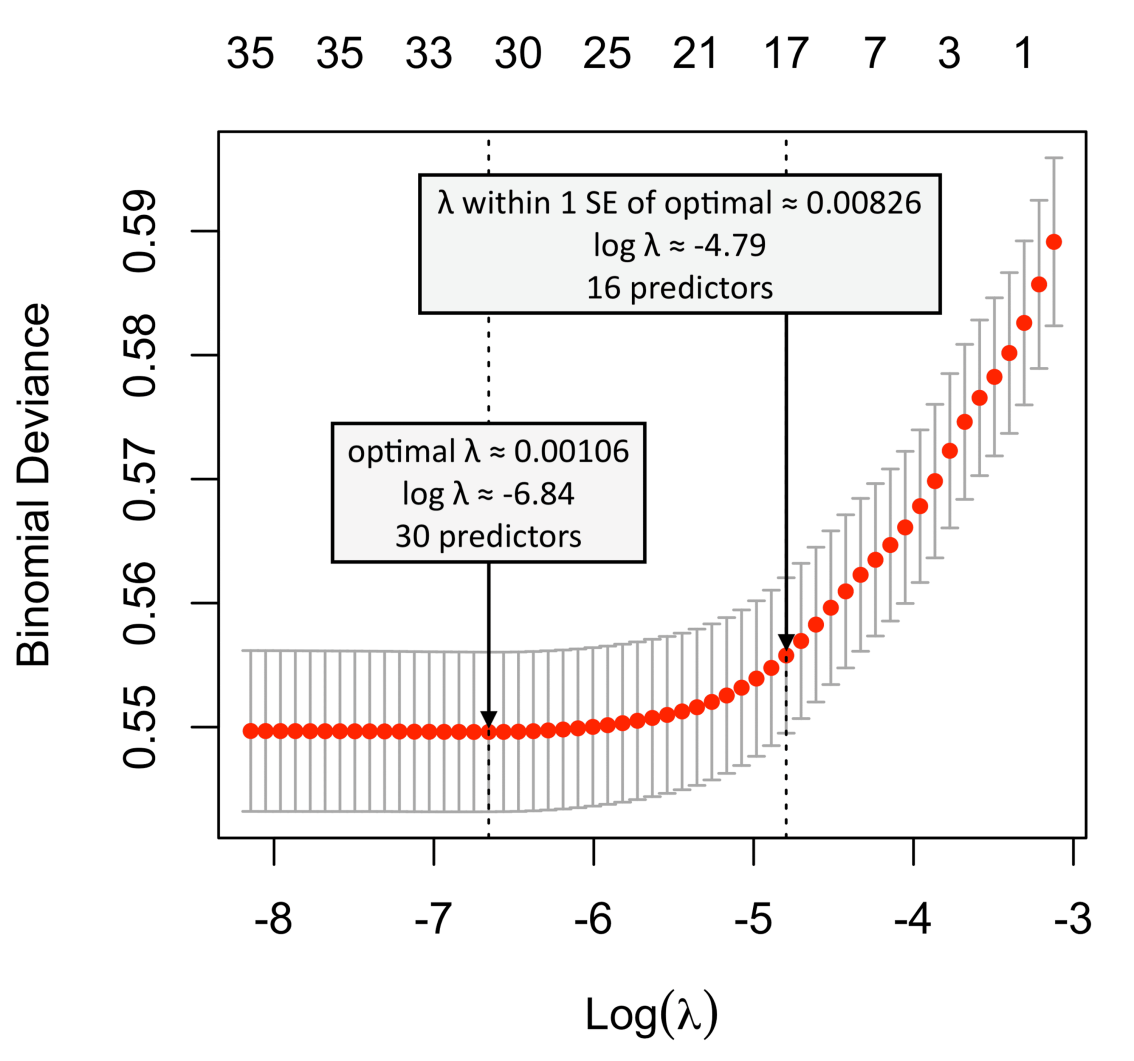
Cross-validation error based on log of the penalty parameter λ The value of 0.00826 was selected to provide the simplest model (16 out of 33 predictors) with acceptable prediction error (within 1 standard error from optimal λ value).

The most impactful predictors of 30-day encounters were Medicaid insurance, followed by inpatient surgery, Medicare, African American race, cardiac comorbidities, obesity, psychiatric disorders, and the Charlson comorbidity score. Table 4 details logistic regression models predicting 30-day postoperative encounters. Both models returned equivalent coefficients. We choose the value of the shrinking parameter λ = 0.00826 (log λ ≈ -4.8). This value is located within 1 standard error of the optimal λ value returned by LASSO (see Figure 3). As a result, it leads to the simplest (kept 16 out of the initial 33 predictors) but still accurate model. Backward stepwise regression kept 19 out of 33 predictors. The rate of missing data in the models was 6.4%. Metrics of models, obtained by 10-fold cross-validation on a 20% sample, are listed in Table 4. Both models were statistically significant (p<0.001) and correctly classified 91.3% cases. The models provided very high specificity and negative predictive value, but low sensitivity (Table 5).

**Table 4:** Logistic regression model predicting presence of 30-day healthcare encounter ^a^ Higher value denotes more significant impact CI: confidence interval; CVA: cerebrovascular incident; GI: gastrointestinal; ICU: intensive care unit; LASSO: least absolute shrinkage and selection operator regression; MIS: minimally invasive surgery

Predictor	LASSO model			Backward elimination model		
	OR (95% CI)	Predictor importance rank^ a^	p-value	OR (95% CI)	Predictor importance rank	p-value
Female gender (ref: male)	1.17 (1.06 - 1.29)	10.7	0.002	1.11 (0.99 - 1.23)	0.0	0.064
African American (ref: other)	1.33 (1.19 - 1.48)	38.4	< 0.001	1.33 (1.20 - 1.48)	38.6	< 0.001
Charlson comorbidity score	1.08 (1.04 - 1.13)	23.7	< 0.001	1.08 (1.04 - 1.13)	21.34	< 0.001
Insurance (ref: private/other)			< 0.001			< 0.001
Medicare	1.42 (1.28 - 1.59)	43.8		1.46 (1.31 - 1.64)	45.7	
Medicaid	2.33 (2.06 - 2.64)	100.0		2.36 (2.08 - 2.68)	100.0	
Lower income quartiles	1.19 (1.06 - 1.25)	18.3	0.002	1.19 (1.08 - 1.34)	17.5	0.001
Urgent/Emergent repair (ref: elective)	1.15 (0.99 - 1.33)	0.0	0.056	1.21 (1.04 - 1.40)	3.5	0.013
Inpatient repair (ref: outpatient)	1.78 (1.50 - 2.06)	54.1	< 0.001	1.73 (1.49 - 2.00)	49.1	< 0.001
Hernia type (ref: groin)	Variables not selected by LASSO					0.001
ventral/umbilical				1.18 (1.06 - 1.32)	5.9	
ventral+groin				1.36 (1.07 - 1.73)	8.8	
MIS approach (ref: open)				1.13 (1.02 - 1.25)	7.2	0.018
Component separation				1.44 (1.11 - 1.87)	4.0	0.007
Cardiac comorbidities	1.32 (1.16 - 1.51)	30.7	< 0.001	1.34 (1.17 - 1.53)	31.4	< 0.001
CVA or hemiparesis/hemiplegia	1.46 (1.20 - 1.78)	12.9	< 0.001	1.46 (1.19 - 1.79)	12.5	< 0.001
Obesity (BMI≥30)	1.34 (1.19 - 1.52)	28.4	< 0.001	1.27 (1.12 - 1.44)	21.33	< 0.001
Cytopenias	1.26 (1.06 - 1.49)	7.7	0.008	1.26 (1.07 - 1.49)	7.4	0.007
Psychiatric disorders	1.34 (1.18 - 1.53)	27.1	< 0.001	1.31 (1.15 - 1.5)	24.9	< 0.001
Chronic pain	1.33 (1.09 - 1.61)	8.9	0.005	1.34 (1.10 - 1.63)	7.5	0.003
Concomitant lysis of adhesions	1.29 (1.04 - 1.6)	9.2	0.019	1.22 (0.98 - 1.51)	4.3	0.077
Concomitant GI or biliary procedure	1.42 (1.09 - 1.85)	10.9	0.009	1.47 (1.13 - 1.92)	11.9	0.005
ICU care	1.38 (1.09 - 1.74)	15.3	0.008	1.35 (1.06 - 1.71)	13.1	0.014

**Table 5:** Models cross-validation and performance. ROC: receiver operating characteristic; PPV: positive predictive value; NPV: negative predictive value

Area under ROC curve	0.697	0.699
Sensitivity	14.3%	11.9%
Specificity	99.8%	99.9%
PPV	46.2%	45.5%
NPV	91.44%	91.42%
Accuracy (95% CI)	91.3% (90.5% - 92.1%)	91.3% (90.5% - 92.1%)

## Discussion

As a vastly prevalent pathology, hernias not only present a surgical challenge but also reveal healthcare disparities. Despite a relative lack of clinical granularity, merged state-level administrative databases cover up to 95% of all HR and all unplanned healthcare encounters. This, to our knowledge, is a novel approach, which provides a real-world picture and accounts for the timeline of these encounters. Understanding of the timeline, diagnostic structure, and predictors of these events will help to develop targeted interventions. For instance, EDVs constituted 75% of postoperative encounters, peaking within the first two days after surgery. Half of these early EDVs were related to GI issues, urinary disturbances, and pain. The latter two conditions were almost always successfully treated in the ED without requiring admission.

Thirty-day readmission rate was 2.3%, which is lower than previously reported. This is likely due to a larger denominator, since we have a more inclusive and comprehensive approach, more representative of standard healthcare settings where primary herniorrhaphies are performed. Readmissions peaked by the end of the first week, primarily due to GI issues and surgical site (SSI) or systemic infections, which also corresponds to previously reported data. Previously reported 30-day ED utilization rates were around 5-6.5%, which aligns with our results (6.4%) [12,16]. Selected previous reports on 30-day readmissions and ED utilization after herniorrhaphy are summarized in Table 6.

**Table 6:** Selected previous reports on 30-day readmissions and ED utilization after herniorrhaphy ^a^ Fraction of all readmissions; ^b^ Geisinger Medical Center healthcare system (central PA) ACS-NSQIP: American College of Surgeons National Surgical Quality Improvement Program; AHR: abdominal hernia repair (includes inguinal/femoral, ventral midline & lateral, incisional); AKI: acute kidney injury;  AMA: against medical advice;  ASA: American Society of Anesthesiology class; CCI: Charlson Comorbidity Index; CCY: cholecystectomy; COPD: chronic obstructive pulmonary disease; CS: component separation; DHD: Danish Hernia Database; DNPR: Danish National Patients Registry; ED: emergency department; GU: genitourinary (urinary tract infection, urinary retention, etc); HR: hernia repair; IHR: inguinal (and femoral) hernia repair; LOS: length of stay; N/A: not available (not reported) data; NRD: National Readmission Database; SOB: shortness of breath; SPARCS: Statewide Planning and Research Cooperative System (New York); SSI: surgical site infection; SSO: surgical site occurrence (seroma, hematoma, wound healing issues); UH: umbilical hernia; VHR: ventral hernia repair.

Reference	Data used	Population	Readmissions	ED visits	Common reasons for readmission^ a^	Predictors of readmissions
Feimster et al., 2020 [1]	NRD, 2013 - 2014	Inpatient VHR ± CS, N=203K	VHR w/o CS: 19.1% (90-d); VHR w/ CS: 22.5% (90-d)	N/A	SSI & systemic infections (32%), SSO (8%), GI issues (6%)	Older age, more comorbidities, obesity, chronic lung disease, Medicaid, lower income, large urban teaching hospital, non-elective surgery, CS vs no CS
Ayuso et al., 2022 [3]	NRD, 2016-2018	Inpatient VHR, N=206K	90-d: 11.3% (MIS); 17.3% (open)	N/A	Infection/sepsis (13.2% MIS – 23.1% open), AKI (6.4% – 7.1%), bowel obstruction (4.2% – 2.6%)	Older age, DM, smoking, LOS, urban teaching hospital, non-elective and open surgery, public insurance
Helgstrand et al., 2013 [7]	DHD + DNPR	Primary M2/M3 and incisional HR, N=6.7K	5.9% (30-d)	N/A	SSI (22%), pain (18.5%), SSO (13.3%), post-op care (5%), cardiac compl. (4.5%), renal/GU (3.5%)	Larger defect, mesh use, tacks, female gender
Brown et al., 2020 [12]	SPARCS, 2005-2014	IHR, N=248K	1.4% (30-d)	5.1% (30-d)	N/A	N/A
Rios-Diaz et al., 2020 [16]	NRD, 2010 - 2014	Inpatient incisional HR, N=16K	7.6% (30-d); 19.4% (1-y)		SSI (25.6%), GI compl. (11%), wound compl. (7.6%), GU (2.6%), respiratory (3.3%), VTE (3%). [1-y data]	Medicare and Medicaid, CCI ≥2, COPD, malignancy, smoking, dependent status, open repair, prolonged LOS
Baltodano et al., 2016 [17]	ACS-NSQIP, 2011-2012	Open VHR, N=17.7K	4.7% (30-d)	N/A	N/A	Inpatient repair, duration of surgery, enterolysis, underweight, diabetes, anemia, LOS, COPD, bleeding disorders, hernia with gangrene, panniculectomy
Mercier et al., 2018 [18]	French National Discharge Database	Non-recurrent IHR, N=123K	3.6 % (30-d)	N/A	Bleeding / GI issues (7%), pain (5.4%), urinary retention (4.9%)	Age, male gender, number of comorbidities, emergency surgery, open approach, concomitant procedures, ICU stay, low hospital surgical volume
Eidelson et al., 2019 [19]	NRD, 2013 - 2014	Inpatient UH, N=102K	8.9% (30-d)	N/A	Infections (25.8%), bowel obstruction (3.2%), AKI (3.1%), PE (2.3%), abdominal pain (1.8%)	Bowel resection, index admission at a for-profit hospital, Medicare, Medicaid, CCI ≥ 2, leaving AMA, elective operation, drug abuse, discharge to a skilled nursing facility
Henriksen et al., 2020 [20]	DHD + DNPR, 2017 - 2018	Small primary VHR, N=1.9K	7% (90-d)	N/A	SSI (17%), abdominal pain (14%), SSO (12%), post-op care (11%), GI issues (7%)	Smoking, BMI >40
Rosero and Joshi, 2020 [21]	ACS-NSQIP, 2012 - 2016	Elective outpatient AHR, N=214K	0.42% (24h)	N/A	Pain (15%), bleeding (14%), GI issues (9%), urinary retention (6.6%), SSI (5.2%)	Age, duration of surgery, dependent functional status, female gender, ASA class, metastatic cancer
Mahan et al., 2020 [22]	Geisinger^b^,2018-2019	Elective CCY (N=2K) and IHR (N=1.6K)	IHR: 1.5% (30-d); 2.5% (90-d)	IHR: 6.5% (30-d); 10.4% (90-d)	Both CCY and IHR.ED: pain (21.6%), urinary (9.7%), SSI/SSO (6.3), SOB (5.5%). Readmission: pain (37.7%), SOB (8.8%), chest pain (7%), SSI/SSO 5.3%	No model provided. 61% 30-d ED visits deemed preventable and 70% deemed related to surgery

SSIs, found to be a leading cause of readmissions, underscore the critical imperative of evidence-based infection control strategies and routine surveillance for early detection and intervention. SSI and other wound-related complications cause half of readmissions within one year after incisional [16] and inguinal HR. Similarly, our study revealed that SSIs were the leading cause (nearly 20%) of all 30-day readmissions. Vigilant monitoring for signs and symptoms of SSI, particularly during the first week post-surgery, allows for prompt initiation of outpatient therapy, including wound care and antibiotics when indicated, to avoid preventable readmissions.

We found that delayed return of bowel function (including constipation, diet intolerance, bowel obstruction, and ileus) was the most common reason for readmission within the first week after herniorrhaphy. Certain ileus-reducing strategies, such as peripherally acting µ-receptor antagonists (alvimopan, methylnaltrexone), demonstrated efficacy in patients undergoing abdominal surgery and using opioid patient-controlled analgesia [23]. Multimodal analgesics, regional anesthesia, and judicious management of volume status can be useful as well.

Inadequate pain control emerged as a primary reason for postoperative EDVs, emphasizing the potential of Enhanced Recovery After Surgery (ERAS) protocols and shared decision-making to optimize pain management. The growing use of ERAS protocols is a hopeful strategy to achieve more reliable pain control postoperatively [24].

Urinary retention was the second most common reason for EDV within the first week of herniorrhaphy. It was almost always successfully treated in the ED and did not require admission. The use of modern anesthetic reversal agents (sugammadex) and the avoidance of general anesthesia when possible [25] are two mitigating strategies to minimize ED visits for postoperative urinary retention.

Predictors of readmissions and EDVs

The most impactful independent predictors of 30-day encounters were Medicaid insurance, followed by inpatient surgery, Medicare, African-American origin, cardiac comorbidities, obesity, psychiatric disorders, and the Charlson comorbidity score. This highlights the significant impact of socio-economic factors on the postoperative course, comparable in magnitude to that of medical comorbidities.

Medicaid patients frequently have higher rates of comorbidities and social determinants that contribute to poor health outcomes. This population is likely to face barriers to accessing elective surgical consultation and proper follow-up care. Disparities based on race, income, and insurance status were observed in readmissions and EDVs, highlighting the need for targeted interventions to address these inequalities. Notably, our study revealed disparities in race, income, and insurance status in both LASSO and backward regression models, adding to a growing body of literature on socioeconomic predictors in hernia outcomes [1,26,27]. The diverse racial and ethnic composition, socioeconomic status, and urban/rural location of the Maryland population allowed us to demonstrate the effect of disparities on readmissions and EDVs. Furthermore, Medicaid and African American heritage were the first and fourth most significant predictors of 30-day encounters, independent of each other. These two populations more frequently have acute presentations requiring emergent surgeries and lower rates of optimal outcomes, leading to almost doubled cumulative costs compared to holders of private insurance [26].

Implementing targeted programs to improve access to elective surgical consultations, enhancing follow-up care, and addressing social determinants of health are critical steps toward achieving equitable surgical outcomes for all patients.

Utilization of EDs

The high utilization of ED services postoperatively prompts consideration of alternative management strategies for complications, potentially reducing unnecessary EDVs. One in 16 patients in our population visited the ED within 30 days after their HR. The overwhelming majority of EDVs in our population included certain diagnostic or therapeutic interventions (Table 3), suggesting that these visits may not always be classified as "unnecessary". EDVs for urinary disorders or acute pain almost never resulted in readmission, indicating that these two causes can be effectively addressed in the ED setting. Brown et al. noted that despite a decrease in readmissions after common general-surgery procedures in recent years, the rate of EDVs has changed minimally [12]. They postulated that this increase could be a consequence of the Hospital Readmission Reduction Program, implemented in 2009. Some patients who historically would be readmitted now undergo workup and management under ED observation. On the other hand, all our patients who visited the ED (by design of SEDD) were discharged home, suggesting that these patients could potentially have been managed in outpatient settings such as a physician's office, urgent care, or by visiting nurses.

Limitations

The main limitation of this study, utilizing administrative databases, is the inability to account for numerous potential clinical predictors of unplanned 30-day encounters. Clinical and demographic heterogeneity also makes it difficult to generate a single universally accurate forecasting model. Accordingly, our model has high specificity but low sensitivity, and does not serve as a screening tool. Rather, based on the diverse Maryland population of all abdominal hernia patients, it captured common risk factors for readmissions and EDVs regardless of hernia type and patient characteristics. However, we realize that state-specific patient mix prevents nationwide generalizations. Our dataset was limited to 2016-2017, and it is possible that there are new unforeseen factors such as limited access to healthcare during the COVID-19 pandemic, improved ERAS protocols, or wider use of telemedicine. However, we believe that the core factors driving unplanned healthcare encounters following HR have remained consistent over time. The nature of state-level databases prevents tracking patients' encounters in neighboring states. State-level HCUP databases also prevent tracking individuals across years, so if the patient underwent an HR in December and got readmitted in January the following year, this encounter could not be captured.

## Conclusions

In this large, state-level analysis linking inpatient, outpatient, and emergency department databases, nearly one in 12 patients experienced an unplanned healthcare encounter within 30 days of hernia repair. EDVs markedly outnumbered readmissions (6.4% vs. 2.3%), most occurring within the first 48 hours and driven primarily by urinary or gastrointestinal complaints and inadequate pain control. Readmissions were largely attributable to SSIs and GI complications. Socioeconomic and insurance-related disparities emerged as the most significant independent predictors of unplanned encounters, underscoring that healthcare access and equity are as critical to postoperative outcomes as medical and surgical factors.

Although the retrospective design and reliance on administrative databases limit clinical granularity and procedure-specific detail, this approach offers a standardized, real-world perspective that equalizes for variations in hernia morphology and surgical technique and reveals risk factors common across a wide spectrum of herniorrhaphies. Our findings highlight the importance of targeted perioperative strategies-including patient education, multimodal analgesia, bowel regimen optimization, infection surveillance, and improved follow-up access-to reduce avoidable postoperative readmissions and EDVs. Future prospective studies integrating greater clinical detail, patient-reported outcomes, and socioeconomic variables are warranted to validate and refine these risk models, ultimately guiding interventions that improve recovery and equity in abdominal wall reconstruction.

## Appendices

**Table 7:** ICD-10-CM and CPT® codes of abdominal wall hernias repairs used in selection of study population ICD-10-CM: International Classification of Diseases, 10th Revision, Procedure Coding System; CPT®: Current Procedural Terminology

ICD-10-CM codes (applicable to inpatient procedures – SID)	
0WQF0ZZ	Repair Abdominal Wall Open Approach
0WQF3ZZ	Repair Abdominal Wall Percutaneous Approach
0WQF4ZZ	Repair Abdominal Wall Percutaneous Endoscopic Approach
0WQFXZ2	Repair Abdominal Wall Stoma External Approach
0WQFXZZ	Repair Abdominal Wall External Approach
0YQ54ZZ	Repair Right Inguinal Region Perc Endo Approach
0YQ60ZZ	Repair Left Inguinal Region Open Approach
0YQ64ZZ	Repair Left Inguinal Region Perc Endo Approach
0YQ70ZZ	Repair Right Femoral Region Open Approach
0YQ74ZZ	Repair Right Femoral Region Perc Endo Approach
0YQ80ZZ	Repair Left Femoral Region Open Approach
0YQ84ZZ	Repair Left Femoral Region Percutaneous Endoscopic Approach
0YQA0ZZ	Repair Bilateral Inguinal Region Open Approach
0YQA4ZZ	Repair Bilateral Inguinal Region Perc Endo Approach
0YQE0ZZ	Repair Bilateral Femoral Region Open Approach
0YQE4ZZ	Repair Bilateral Femoral Region Perc Endo Approach
0YU507Z	Supplement R Inguinal Region with Autol Sub Open Approach
0YU50JZ	Supplement R Inguinal Region with Synth Sub Open Approach
0YU50KZ	Supplement R Inguinal Region with Nonaut Sub Open Approach
0YU547Z	Supplement R Inguinal Region w Autol Sub Perc Endo
0YU54JZ	Supplement R Inguinal Region w Synth Sub Perc Endo
0YU54KZ	Supplement R Inguinal Region w Nonaut Sub Perc Endo
0YU607Z	Supplement L Inguinal Region with Autol Sub Open Approach
0YU60JZ	Supplement L Inguinal Region with Synth Sub Open Approach
0YU60KZ	Supplement L Inguinal Region with Nonaut Sub Open Approach
0YU647Z	Supplement L Inguinal Region w Autol Sub Perc Endo
0YU64JZ	Supplement L Inguinal Region w Synth Sub Perc Endo
0YU64KZ	Supplement L Inguinal Region w Nonaut Sub Perc Endo
0YU707Z	Supplement R Femoral Region with Autol Sub Open Approach
0YU70JZ	Supplement R Femoral Region with Synth Sub Open Approach
0YU70KZ	Supplement R Femoral Region with Nonaut Sub Open Approach
0YU747Z	Supplement R Femoral Region w Autol Sub Perc Endo
0YU74JZ	Supplement R Femoral Region w Synth Sub Perc Endo
0YU74KZ	Supplement R Femoral Region w Nonaut Sub Perc Endo
0YU807Z	Supplement Left Femoral Region with Autol Sub Open Approach
0YU80JZ	Supplement Left Femoral Region with Synth Sub Open Approach
0YU80KZ	Supplement L Femoral Region with Nonaut Sub Open Approach
0YU847Z	Supplement L Femoral Region w Autol Sub Perc Endo
0YU84JZ	Supplement L Femoral Region w Synth Sub Perc Endo
0YU84KZ	Supplement L Femoral Region w Nonaut Sub Perc Endo
0YUA07Z	Supplement Bi Inguinal Region with Autol Sub Open Approach
0YUA0JZ	Supplement Bi Inguinal Region with Synth Sub Open Approach
0YUA0KZ	Supplement Bi Inguinal Region with Nonaut Sub Open Approach
0YUA47Z	Supplement Bi Inguinal Region w Autol Sub Perc Endo
0YUA4JZ	Supplement Bi Inguinal Region w Synth Sub Perc Endo
0YUA4KZ	Supplement Bi Inguinal Region w Nonaut Sub Perc Endo
0YUE07Z	Supplement Bi Femoral Region with Autol Sub Open Approach
0YUE0JZ	Supplement Bi Femoral Region with Synth Sub Open Approach
0YUE0KZ	Supplement Bi Femoral Region with Nonaut Sub Open Approach
0YUE47Z	Supplement Bi Femoral Region w Autol Sub Perc Endo
0YUE4JZ	Supplement Bi Femoral Region w Synth Sub Perc Endo
0YUE4KZ	Supplement Bi Femoral Region w Nonaut Sub Perc Endo
0WUF07Z	Supplement Abdominal Wall with Autol Sub Open Approach
0WUF0JZ	Supplement Abdominal Wall with Synth Sub Open Approach
0WUF0KZ	Supplement Abdominal Wall with Nonaut Sub Open Approach
0WUF47Z	Supplement Abdominal Wall with Autol Sub Perc Endo Approach
0WUF4JZ	Supplement Abdominal Wall with Synth Sub Perc Endo Approach
0WUF4KZ	Supplement Abd Wall with Nonaut Sub Perc Endo Approach
0KNK0ZZ	Release Right Abdomen Muscle, Open Approach
0KNK3ZZ	Release Right Abdomen Muscle, Percutaneous Approach
0KNK4ZZ	Release Right Abdomen Muscle, Percutaneous Endoscopic Approach
0KNKXZZ	Release Right Abdomen Muscle, External Approach
0KNL0ZZ	Release Left Abdomen Muscle, Open Approach
0KNL3ZZ	Release Left Abdomen Muscle, Percutaneous Approach
0KNL4ZZ	Release Left Abdomen Muscle, Percutaneous Endoscopic Approach
0KNLXZZ	Release Left Abdomen Muscle, External Approach
CPT® codes (applicable to outpatient procedures – SASD)	
49491	Repair, initial inguinal hernia, preterm infant (younger than 37 weeks gestation at birth), performed from birth up to 50 weeks postconception age, with or without hydrocelectomy; reducible
49492	Repair, initial inguinal hernia, preterm infant (younger than 37 weeks gestation at birth), performed from birth up to 50 weeks postconception age, with or without hydrocelectomy; incarcerated or strangulated
49495	Repair, initial inguinal hernia, full term infant younger than age 6 months, or preterm infant older than 50 weeks postconception age and younger than age 6 months at the time of surgery, with or without hydrocelectomy; reducible
49496	Repair, initial inguinal hernia, full term infant younger than age 6 months, or preterm infant older than 50 weeks postconception age and younger than age 6 months at the time of surgery, with or without hydrocelectomy; incarcerated or strangulated
49500	Repair initial inguinal hernia, age 6 months to younger than 5 years, with or without hydrocelectomy; reducible
49501	Repair initial inguinal hernia, age 6 months to younger than 5 years, with or without hydrocelectomy; incarcerated or strangulated
49505	Repair initial inguinal hernia, age 5 years or older; reducible
49507	Repair initial inguinal hernia, age 5 years or older; incarcerated or strangulated
49520	Repair recurrent inguinal hernia, any age; reducible
49521	Repair recurrent inguinal hernia, any age; incarcerated or strangulated
49525	Repair inguinal hernia, sliding, any age
49540	Repair lumbar hernia
49550	Repair initial femoral hernia, any age; reducible
49553	Repair initial femoral hernia, any age; incarcerated or strangulated
49555	Repair recurrent femoral hernia; reducible
49557	Repair recurrent femoral hernia; incarcerated or strangulated
49560	Repair initial incisional or ventral hernia; reducible
49561	Repair initial incisional or ventral hernia; incarcerated or strangulated
49565	Repair recurrent incisional or ventral hernia; reducible
49566	Repair recurrent incisional or ventral hernia; incarcerated or strangulated
49570	Repair epigastric hernia (eg, preperitoneal fat); reducible (separate procedure)
49572	Repair epigastric hernia (eg, preperitoneal fat); incarcerated or strangulated
49580	Repair umbilical hernia, younger than age 5 years; reducible
49582	Repair umbilical hernia, younger than age 5 years; incarcerated or strangulated
49585	Repair umbilical hernia, age 5 years or older; reducible
49587	Repair umbilical hernia, age 5 years or older; incarcerated or strangulated
49590	Repair spigelian hernia
49650	Laparoscopy, surgical; repair initial inguinal hernia
49651	Laparoscopy, surgical; repair recurrent inguinal hernia
49652	Laparoscopy, surgical, repair, ventral, umbilical, spigelian or epigastric hernia (includes mesh insertion, when performed); reducible
49653	Laparoscopy, surgical, repair, ventral, umbilical, spigelian or epigastric hernia (includes mesh insertion, when performed); incarcerated or strangulated
49654	Laparoscopy, surgical, repair, incisional hernia (includes mesh insertion, when performed); reducible
49655	Laparoscopy, surgical, repair, incisional hernia (includes mesh insertion, when performed); incarcerated or strangulated
49656	Laparoscopy, surgical, repair, recurrent incisional hernia (includes mesh insertion, when performed); reducible
49657	Laparoscopy, surgical, repair, recurrent incisional hernia (includes mesh insertion, when performed); incarcerated or strangulated
15734	Muscle, myocutaneous, or fasciocutaneous flap; trunk
49568	Implantation of mesh or other prosthesis for open incisional or ventral hernia repair or mesh for closure of debridement for necrotizing soft tissue infection (List separately in addition to code for the incisional or ventral hernia repair) (Use 49568 in conjunction with 11004–11006, 49560–49566)
49659	Unlisted laparoscopy procedure, hernioplasty, herniorrhaphy, herniotomy

**Table 8:** ICD-10-CM diagnostic codes for abdominal wall hernias used in selection of study population ICD-10-CM: International Classification of Diseases, 10th Revision, Procedure Coding System

K4000	Bilateral inguinal hernia with obstruction without gangrene not specified as recurrent
K4001	Bilateral inguinal hernia with obstruction without gangrene recurrent
K4010	Bilateral inguinal hernia with gangrene not specified as recurrent
K4011	Bilateral inguinal hernia with gangrene recurrent
K4020	Bilateral inguinal hernia without obstruction or gangrene not specified as recurrent
K4021	Bilateral inguinal hernia without obstruction or gangrene recurrent
K4030	Unilateral inguinal hernia with obstruction without gangrene not specified as recurrent
K4031	Unilateral inguinal hernia with obstruction without gangrene recurrent
K4040	Unilateral inguinal hernia with gangrene not specified as recurrent
K4041	Unilateral inguinal hernia with gangrene recurrent
K4090	Unilateral inguinal hernia without obstruction or gangrene not specified as recurrent
K4091	Unilateral inguinal hernia without obstruction or gangrene recurrent
K4100	Bilateral femoral hernia with obstruction without gangrene not specified as recurrent
K4101	Bilateral femoral hernia with obstruction without gangrene recurrent
K4110	Bilateral femoral hernia with gangrene not specified as recurrent
K4111	Bilateral femoral hernia with gangrene recurrent
K4120	Bilateral femoral hernia without obstruction or gangrene not specified as recurrent
K4121	Bilateral femoral hernia without obstruction or gangrene recurrent
K4130	Unilateral femoral hernia with obstruction without gangrene not specified as recurrent
K4131	Unilateral femoral hernia with obstruction without gangrene recurrent
K4140	Unilateral femoral hernia with gangrene not specified as recurrent
K4141	Unilateral femoral hernia with gangrene recurrent
K4190	Unilateral femoral hernia without obstruction or gangrene not specified as recurrent
K4191	Unilateral femoral hernia without obstruction or gangrene recurrent
K420	Umbilical hernia with obstruction without gangrene
K421	Umbilical hernia with gangrene
K429	Umbilical hernia without obstruction or gangrene
K430	Incisional hernia with obstruction without gangrene
K431	Incisional hernia with gangrene
K432	Incisional hernia without obstruction or gangrene
K433	Parastomal hernia with obstruction without gangrene
K434	Parastomal hernia with gangrene
K435	Parastomal hernia without obstruction or gangrene
K436	Other and unspecified ventral hernia with obstruction without gangrene
K437	Other and unspecified ventral hernia with gangrene
K439	Ventral hernia without obstruction or gangrene
K450	Other specified abdominal hernia with obstruction without gangrene
K451	Other specified abdominal hernia with gangrene
K458	Other specified abdominal hernia without obstruction or gangrene
K460	Unspecified abdominal hernia with obstruction without gangrene
K461	Unspecified abdominal hernia with gangrene
K469	Unspecified abdominal hernia without obstruction or gangrene

**Table 9:** Primary diagnoses (ICD-10-CM) of postoperative ED visits or readmissions ICD-10-CM: International Classification of Diseases, 10th Revision, Procedure Coding System

Postoperative pain	
G8918	Other acute postprocedural pain
N50811	Right testicular pain
N50812	Left testicular pain
N5082	Scrotal pain
R1010	Upper abdominal pain unspecified
R1011	Right upper quadrant pain
R1012	Left upper quadrant pain
R1013	Epigastric pain
R102	Pelvic and perineal pain
R1030	Lower abdominal pain unspecified
R1031	Right lower quadrant pain
R1032	Left lower quadrant pain
R1033	Periumbilical pain
R1084	Generalized abdominal pain
R109	Unspecified abdominal pain
T85848A	Pain due to other internal prosthetic devices implants and grafts initial encounter
Urinary complaints/complications	
N3000	Acute cystitis without hematuria
N3001	Acute cystitis with hematuria
N329	Bladder disorder unspecified
N390	Urinary tract infection site not specified
N401	Benign prostatic hyperplasia with lower urinary tract symptoms
N5089	Other specified disorders of the male genital organs
N99114	Postprocedural urethral stricture male unspecified
N9989	Other postprocedural complications and disorders of genitourinary system
R300	Dysuria
R310	Gross hematuria
R319	Hematuria unspecified
R330	Drug induced retention of urine
R338	Other retention of urine
R339	Retention of urine unspecified
R350	Frequency of micturition
R3915	Urgency of urination
T83021A	Displacement of indwelling urethral catheter initial encounter
T83028A	Displacement of other urinary catheter initial encounter
T83038A	Leakage of other urinary catheter initial encounter
T83098A	Other mechanical complication of other urinary catheter initial encounter
T83511A	Infection and inflammatory reaction due to indwelling urethral catheter initial encounter
T83518A	Infection and inflammatory reaction due to other urinary catheter initial encounter
T8351XA	Infection and inflammatory reaction due to indwelling urinary catheter initial encounter
Z466	Encounter for fitting and adjustment of urinary device
Delayed return of bowel function, constipation, nausea/emesis	
K5641	Fecal impaction
K5649	Other impaction of intestine
K565	Intestinal adhesions [bands] with obstruction (postprocedural) (postinfection)
K5650	Intestinal adhesions [bands] unspecified as to partial versus complete obstruction
K5651	Intestinal adhesions [bands] with partial obstruction
K5660	Unspecified intestinal obstruction
K56600	Partial intestinal obstruction unspecified as to cause
K56609	Unspecified intestinal obstruction unspecified as to partial versus complete obstruction
K5669	Other intestinal obstruction
K56699	Other intestinal obstruction unspecified as to partial versus complete
K567	Ileus unspecified
K5900	Constipation unspecified
K5901	Slow transit constipation
K5903	Drug induced constipation
K5909	Other constipation
K913	Postprocedural intestinal obstruction
K9130	Postprocedural intestinal obstruction unspecified as to partial versus complete
K9131	Postprocedural partial intestinal obstruction
R110	Nausea
R1110	Vomiting unspecified
R1111	Vomiting without nausea
R1112	Projectile vomiting
R112	Nausea with vomiting unspecified
R140	Abdominal distension (gaseous)
Surgical site hematoma/seroma	
L7621	Postprocedural hemorrhage of skin and subcutaneous tissue following a dermatologic procedure
L7622	Postprocedural hemorrhage of skin and subcutaneous tissue following other procedure
L7632	Postprocedural hematoma of skin and subcutaneous tissue following other procedure
L7633	Postprocedural seroma of skin and subcutaneous tissue following a dermatologic procedure
L7634	Postprocedural seroma of skin and subcutaneous tissue following other procedure
L7682	Other postprocedural complications of skin and subcutaneous tissue
M7981	Nontraumatic hematoma of soft tissue
M96830	Postprocedural hemorrhage of a musculoskeletal structure following a musculoskeletal system procedure
M96831	Postprocedural hemorrhage of a musculoskeletal structure following other procedure
M96840	Postprocedural hematoma of a musculoskeletal structure following a musculoskeletal system procedure
M96842	Postprocedural seroma of a musculoskeletal structure following a musculoskeletal system procedure
M96843	Postprocedural seroma of a musculoskeletal structure following other procedure
N99820	Postprocedural hemorrhage of a genitourinary system organ or structure following a genitourinary system procedure
N99821	Postprocedural hemorrhage of a genitourinary system organ or structure following other procedure
N99840	Postprocedural hematoma of a genitourinary system organ or structure following a genitourinary system procedure
N99841	Postprocedural hematoma of a genitourinary system organ or structure following other procedure
R58	Hemorrhage not elsewhere classified
T8583XA	Hemorrhage due to internal prosthetic devices implants and grafts not elsewhere classified initial encounter
Surgical site infection	
L02211	Cutaneous abscess of abdominal wall
L02214	Cutaneous abscess of groin
L02216	Cutaneous abscess of umbilicus
L0291	Cutaneous abscess unspecified
L03311	Cellulitis of abdominal wall
L03314	Cellulitis of groin
L0390	Cellulitis unspecified
L089	Local infection of the skin and subcutaneous tissue unspecified
T814XXA	Infection following a procedure initial encounter
T814XXD	Infection following a procedure subsequent encounter
T8579XA	Infection and inflammatory reaction due to other internal prosthetic devices implants and grafts initial encounter
Cardiac complaints/complications	
I110	Hypertensive heart disease with heart failure
I130	Hypertensive heart and chronic kidney disease with heart failure and stage 1 through stage 4 chronic kidney disease or unspecified chronic kidney disease
I132	Hypertensive heart and chronic kidney disease with heart failure and with stage 5 chronic kidney disease or end stage renal disease
I2119	ST elevation (STEMI) myocardial infarction involving other coronary artery of inferior wall
I213	ST elevation (STEMI) myocardial infarction of unspecified site
I214	Non-ST elevation (NSTEMI) myocardial infarction
I2510	Atherosclerotic heart disease of native coronary artery without angina pectoris
I25110	Atherosclerotic heart disease of native coronary artery with unstable angina pectoris
I441	Atrioventricular block second degree
I469	Cardiac arrest cause unspecified
I471	Supraventricular tachycardia
I472	Ventricular tachycardia
I480	Paroxysmal atrial fibrillation
I481	Persistent atrial fibrillation
I482	Chronic atrial fibrillation
I483	Typical atrial flutter
I4891	Unspecified atrial fibrillation
I4892	Unspecified atrial flutter
I4901	Ventricular fibrillation
I495	Sick sinus syndrome
I499	Cardiac arrhythmia unspecified
I5023	Acute on chronic systolic (congestive) heart failure
I5032	Chronic diastolic (congestive) heart failure
I5033	Acute on chronic diastolic (congestive) heart failure
I5042	Chronic combined systolic (congestive) and diastolic (congestive) heart failure
I5043	Acute on chronic combined systolic (congestive) and diastolic (congestive) heart failure
I509	Heart failure unspecified
R000	Tachycardia unspecified
R001	Bradycardia unspecified
R002	Palpitations
Respiratory complaints/complications	
J13	Pneumonia due to* Streptococcus pneumoniae*
J159	Unspecified bacterial pneumonia
J168	Pneumonia due to other specified infectious organisms
J181	Lobar pneumonia unspecified organism
J188	Other pneumonia unspecified organism
J189	Pneumonia unspecified organism
J208	Acute bronchitis due to other specified organisms
J209	Acute bronchitis unspecified
J40	Bronchitis not specified as acute or chronic
J440	Chronic obstructive pulmonary disease with acute lower respiratory infection
J441	Chronic obstructive pulmonary disease with (acute) exacerbation
J449	Chronic obstructive pulmonary disease unspecified
J4551	Severe persistent asthma with (acute) exacerbation
J4541	Moderate persistent asthma with (acute) exacerbation
J45901	Unspecified asthma with (acute) exacerbation
J690	Pneumonitis due to inhalation of food and vomit
J90	Pleural effusion not elsewhere classified
J939	Pneumothorax unspecified
J95811	Postprocedural pneumothorax
J9589	Other postprocedural complications and disorders of respiratory system not elsewhere classified
J9601	Acute respiratory failure with hypoxia
J9610	Chronic respiratory failure unspecified whether with hypoxia or hypercapnia
J9621	Acute and chronic respiratory failure with hypoxia
J9690	Respiratory failure unspecified unspecified whether with hypoxia or hypercapnia
J9811	Atelectasis
R0600	Dyspnea unspecified
R0602	Shortness of breath
R0609	Other forms of dyspnea
"Surgical aftercare" including management of drains or dressings	
Z4801	Encounter for change or removal of surgical wound dressing
Z4802	Encounter for removal of sutures
Z4803	Encounter for change or removal of drains
Z48815	Encounter for surgical aftercare following surgery on the digestive system
Z48817	Encounter for surgical aftercare following surgery on the skin and subcutaneous tissue
Z4889	Encounter for other specified surgical aftercare
Wound separation or dehiscence	
T8130XA	Disruption of wound unspecified initial encounter
T8131XA	Disruption of external operation (surgical) wound not elsewhere classified initial encounter
T8132XA	Disruption of internal operation (surgical) wound not elsewhere classified initial encounter
Fluid and electrolyte disturbances	
E8342	Hypomagnesemia
E8350	Unspecified disorder of calcium metabolism
E860	Dehydration
E861	Hypovolemia
E871	Hypo-osmolality and hyponatremia
E872	Acidosis
E875	Hyperkalemia
E876	Hypokalemia
E8770	Fluid overload unspecified
R571	Hypovolemic shock
Other gastrointestinal complications, including bleeding	
K91840	Postprocedural hemorrhage of a digestive system organ or structure following a digestive system procedure
K91841	Postprocedural hemorrhage of a digestive system organ or structure following other procedure
K91870	Postprocedural hematoma of a digestive system organ or structure following a digestive system procedure
K91871	Postprocedural hematoma of a digestive system organ or structure following other procedure
K91872	Postprocedural seroma of a digestive system organ or structure following a digestive system procedure
K91873	Postprocedural seroma of a digestive system organ or structure following other procedure
K9189	Other postprocedural complications and disorders of digestive system
Hernias	
K4030	Unilateral inguinal hernia with obstruction without gangrene not specified as recurrent
K4090	Unilateral inguinal hernia without obstruction or gangrene not specified as recurrent
K4191	Unilateral femoral hernia without obstruction or gangrene recurrent
K420	Umbilical hernia with obstruction without gangrene
K429	Umbilical hernia without obstruction or gangrene
K430	Incisional hernia with obstruction without gangrene
K432	Incisional hernia without obstruction or gangrene
K436	Other and unspecified ventral hernia with obstruction without gangrene
K439	Ventral hernia without obstruction or gangrene
K449	Diaphragmatic hernia without obstruction or gangrene
K458	Other specified abdominal hernia without obstruction or gangrene
K460	Unspecified abdominal hernia with obstruction without gangrene
K469	Unspecified abdominal hernia without obstruction or gangrene
Sepsis and systemic infection	
A401	Sepsis due to streptococcus group B
A4151	Sepsis due to *Escherichia coli*
A4152	Sepsis due to *Pseudomonas*
A4159	Other gram-negative sepsis
A4181	Sepsis due to *Enterococcus*
A4189	Other specified sepsis
A419	Sepsis unspecified organism
A4901	Methicillin susceptible *Staphylococcus aureus* infection unspecified site
R5082	Postprocedural fever
R509	Fever unspecified
R6521	Severe sepsis with septic shock
Venous thromboembolism (DVT/PE)	
I2609	Other pulmonary embolism with acute cor pulmonale
I2692	Saddle embolus of pulmonary artery without acute cor pulmonale
I2699	Other pulmonary embolism without acute cor pulmonale
I8001	Phlebitis and thrombophlebitis of superficial vessels of right lower extremity
I8002	Phlebitis and thrombophlebitis of superficial vessels of left lower extremity
I808	Phlebitis and thrombophlebitis of other sites
I82402	Acute embolism and thrombosis of unspecified deep veins of left lower extremity
I82411	Acute embolism and thrombosis of right femoral vein
I82412	Acute embolism and thrombosis of left femoral vein
I82432	Acute embolism and thrombosis of left popliteal vein
I82441	Acute embolism and thrombosis of right tibial vein
I82492	Acute embolism and thrombosis of other specified deep vein of left lower extremity
I824Z2	Acute embolism and thrombosis of unspecified deep veins of left distal lower extremity
I824Z9	Acute embolism and thrombosis of unspecified deep veins of unspecified distal lower extremity
I82819	Embolism and thrombosis of superficial veins of unspecified lower extremity
Dizziness, syncope, malaise	
R42	Dizziness and giddiness
R531	Weakness
R5381	Other malaise
R5383	Other fatigue
R55	Syncope and collapse

**Table 10:** Diagnostic and procedural codes (ICD-10) associated with presence of postoperative readmission or ED visit within 30 days ICD-10: International Classification of Diseases, 10th Revision

ICD-10-CM diagnostic codes	
A047	Enterocolitis due to Clostridium difficile
A419	Sepsis unspecified organism
B001	Herpesviral vesicular dermatitis
B182	Chronic viral hepatitis C
B1920	Unspecified viral hepatitis C without hepatic coma
B370	Candidal stomatitis
B952	Enterococcus as the cause of diseases classified elsewhere
B9562	Methicillin resistant Staphylococcus aureus infection as the cause of diseases classified elsewhere
B9620	Unspecified Escherichia coli [E. coli] as the cause of diseases classified elsewhere
B964	Proteus (mirabilis) (morganii) as the cause of diseases classified elsewhere
B9689	Other specified bacterial agents as the cause of diseases classified elsewhere
C259	Malignant neoplasm of pancreas unspecified
C785	Secondary malignant neoplasm of large intestine and rectum
C786	Secondary malignant neoplasm of retroperitoneum and peritoneum
D170	Benign lipomatous neoplasm of skin and subcutaneous tissue of head face and neck
D490	Neoplasm of unspecified behavior of digestive system
D509	Iron deficiency anemia unspecified
D539	Nutritional anemia unspecified
D573	Sickle-cell trait
D61818	Other pancytopenia
D62	Acute posthemorrhagic anemia
D631	Anemia in chronic kidney disease
D638	Anemia in other chronic diseases classified elsewhere
D649	Anemia unspecified
D6959	Other secondary thrombocytopenia
D696	Thrombocytopenia unspecified
D72828	Other elevated white blood cell count
D72829	Elevated white blood cell count unspecified
D8689	Sarcoidosis of other sites
E038	Other specified hypothyroidism
E1042	Type 1 diabetes mellitus with diabetic polyneuropathy
E1121	Type 2 diabetes mellitus with diabetic nephropathy
E1122	Type 2 diabetes mellitus with diabetic chronic kidney disease
E1140	Type 2 diabetes mellitus with diabetic neuropathy unspecified
E1142	Type 2 diabetes mellitus with diabetic polyneuropathy
E1152	Type 2 diabetes mellitus with diabetic peripheral angiopathy with gangrene
E11649	Type 2 diabetes mellitus with hypoglycemia without coma
E1165	Type 2 diabetes mellitus with hyperglycemia
E118	Type 2 diabetes mellitus with unspecified complications
E271	Primary adrenocortical insufficiency
E43	Unspecified severe protein-calorie malnutrition
E441	Mild protein-calorie malnutrition
E46	Unspecified protein-calorie malnutrition
E6601	Morbid (severe) obesity due to excess calories
E662	Morbid (severe) obesity with alveolar hypoventilation
E663	Overweight
E781	Pure hyperglyceridemia
E8339	Other disorders of phosphorus metabolism
E8342	Hypomagnesemia
E8351	Hypocalcemia
E8352	Hypercalcemia
E854	Organ-limited amyloidosis
E860	Dehydration
E861	Hypovolemia
E870	Hyperosmolality and hypernatremia
E871	Hypo-osmolality and hyponatremia
E872	Acidosis
E873	Alkalosis
E874	Mixed disorder of acid-base balance
E875	Hyperkalemia
E876	Hypokalemia
E8770	Fluid overload unspecified
E8809	Other disorders of plasma-protein metabolism not elsewhere classified
F0390	Unspecified dementia without behavioral disturbance
F05	Delirium due to known physiological condition
F1010	Alcohol abuse uncomplicated
F1020	Alcohol dependence uncomplicated
F1021	Alcohol dependence in remission
F10239	Alcohol dependence with withdrawal unspecified
F1120	Opioid dependence uncomplicated
F1420	Cocaine dependence uncomplicated
F1490	Cocaine use unspecified uncomplicated
F17210	Nicotine dependence cigarettes uncomplicated
F1990	Other psychoactive substance use unspecified uncomplicated
F209	Schizophrenia unspecified
F259	Schizoaffective disorder unspecified
F3130	Bipolar disorder current episode depressed mild or moderate severity unspecified
F319	Bipolar disorder unspecified
F329	Major depressive disorder single episode unspecified
F332	Major depressive disorder recurrent severe without psychotic features
F339	Major depressive disorder recurrent unspecified
F419	Anxiety disorder unspecified
F4310	Post-traumatic stress disorder unspecified
G43909	Migraine unspecified not intractable without status migrainosus
G4700	Insomnia unspecified
G4733	Obstructive sleep apnea (adult) (pediatric)
G5601	Carpal tunnel syndrome right upper limb
G8918	Other acute postprocedural pain
G8929	Other chronic pain
G919	Hydrocephalus unspecified
G9341	Metabolic encephalopathy
H409	Unspecified glaucoma
H43819	Vitreous degeneration unspecified eye
H578	Other specified disorders of eye and adnexa
H8109	Menieres disease unspecified ear
H9190	Unspecified hearing loss unspecified ear
I071	Rheumatic tricuspid insufficiency
I081	Rheumatic disorders of both mitral and tricuspid valves
I083	Combined rheumatic disorders of mitral aortic and tricuspid valves
I110	Hypertensive heart disease with heart failure
I120	Hypertensive chronic kidney disease with stage 5 chronic kidney disease or end stage renal disease
I129	Hypertensive chronic kidney disease with stage 1 through stage 4 chronic kidney disease or unspecified chronic kidney disease
I2510	Atherosclerotic heart disease of native coronary artery without angina pectoris
I25119	Atherosclerotic heart disease of native coronary artery with unspecified angina pectoris
I252	Old myocardial infarction
I255	Ischemic cardiomyopathy
I272	Other secondary pulmonary hypertension
I428	Other cardiomyopathies
I43	Cardiomyopathy in diseases classified elsewhere
I440	Atrioventricular block first degree
I471	Supraventricular tachycardia
I480	Paroxysmal atrial fibrillation
I482	Chronic atrial fibrillation
I4891	Unspecified atrial fibrillation
I5021	Acute systolic (congestive) heart failure
I5022	Chronic systolic (congestive) heart failure
I5030	Unspecified diastolic (congestive) heart failure
I5032	Chronic diastolic (congestive) heart failure
I509	Heart failure unspecified
I69351	Hemiplegia and hemiparesis following cerebral infarction affecting right dominant side
I69354	Hemiplegia and hemiparesis following cerebral infarction affecting left non-dominant side
I69398	Other sequelae of cerebral infarction
I739	Peripheral vascular disease unspecified
I82431	Acute embolism and thrombosis of right popliteal vein
I8390	Asymptomatic varicose veins of unspecified lower extremity
I878	Other specified disorders of veins
I952	Hypotension due to drugs
I953	Hypotension of hemodialysis
I9581	Postprocedural hypotension
I959	Hypotension unspecified
J156	Pneumonia due to other Gram-negative bacteria
J189	Pneumonia unspecified organism
J40	Bronchitis not specified as acute or chronic
J441	Chronic obstructive pulmonary disease with (acute) exacerbation
J449	Chronic obstructive pulmonary disease unspecified
J45901	Unspecified asthma with (acute) exacerbation
J690	Pneumonitis due to inhalation of food and vomit
J811	Chronic pulmonary edema
J8410	Pulmonary fibrosis unspecified
J90	Pleural effusion not elsewhere classified
J942	Hemothorax
J95821	Acute postprocedural respiratory failure
J9601	Acute respiratory failure with hypoxia
J9610	Chronic respiratory failure unspecified whether with hypoxia or hypercapnia
J9611	Chronic respiratory failure with hypoxia
J9811	Atelectasis
K228	Other specified diseases of esophagus
K2900	Acute gastritis without bleeding
K3184	Gastroparesis
K4000	Bilateral inguinal hernia with obstruction without gangrene not specified as recurrent
K4031	Unilateral inguinal hernia with obstruction without gangrene recurrent
K4040	Unilateral inguinal hernia with gangrene not specified as recurrent
K4090	Unilateral inguinal hernia without obstruction or gangrene not specified as recurrent
K430	Incisional hernia with obstruction without gangrene
K432	Incisional hernia without obstruction or gangrene
K433	Parastomal hernia with obstruction without gangrene
K435	Parastomal hernia without obstruction or gangrene
K440	Diaphragmatic hernia with obstruction without gangrene
K449	Diaphragmatic hernia without obstruction or gangrene
K450	Other specified abdominal hernia with obstruction without gangrene
K458	Other specified abdominal hernia without obstruction or gangrene
K5090	Crohns disease unspecified without complications
K521	Toxic gastroenteritis and colitis
K550	Acute vascular disorders of intestine
K559	Vascular disorder of intestine unspecified
K565	Intestinal adhesions [bands] with obstruction (postprocedural) (postinfection)
K5660	Unspecified intestinal obstruction
K5669	Other intestinal obstruction
K567	Ileus unspecified
K5790	Diverticulosis of intestine part unspecified without perforation or abscess without bleeding
K589	Irritable bowel syndrome without diarrhea
K5900	Constipation unspecified
K5901	Slow transit constipation
K598	Other specified functional intestinal disorders
K631	Perforation of intestine (nontraumatic)
K632	Fistula of intestine
K6389	Other specified diseases of intestine
K651	Peritoneal abscess
K660	Peritoneal adhesions (postprocedural) (postinfection)
K661	Hemoperitoneum
K7030	Alcoholic cirrhosis of liver without ascites
K7290	Hepatic failure unspecified without coma
K7460	Unspecified cirrhosis of liver
K766	Portal hypertension
K8020	Calculus of gallbladder without cholecystitis without obstruction
K8080	Other cholelithiasis without obstruction
K810	Acute cholecystitis
K811	Chronic cholecystitis
K8590	Acute pancreatitis without necrosis or infection unspecified
K861	Other chronic pancreatitis
K911	Postgastric surgery syndromes
K913	Postprocedural intestinal obstruction
K9181	Other intraoperative complications of digestive system
K91841	Postprocedural hemorrhage of a digestive system organ or structure following other procedure
K91870	Postprocedural hematoma of a digestive system organ or structure following a digestive system procedure
K9189	Other postprocedural complications and disorders of digestive system
K921	Melena
K922	Gastrointestinal hemorrhage unspecified
K9423	Gastrostomy malfunction
L02211	Cutaneous abscess of abdominal wall
L03115	Cellulitis of right lower limb
L03311	Cellulitis of abdominal wall
L299	Pruritus unspecified
L7621	Postprocedural hemorrhage of skin and subcutaneous tissue following a dermatologic procedure
L7622	Postprocedural hemorrhage of skin and subcutaneous tissue following other procedure
L83	Acanthosis nigricans
L89152	Pressure ulcer of sacral region stage 2
L89153	Pressure ulcer of sacral region stage 3
L89620	Pressure ulcer of left heel unstageable
L910	Hypertrophic scar
M109	Gout unspecified
M13869	Other specified arthritis unspecified knee
M25512	Pain in left shoulder
M329	Systemic lupus erythematosus unspecified
M359	Systemic involvement of connective tissue unspecified
M4186	Other forms of scoliosis lumbar region
M542	Cervicalgia
M549	Dorsalgia unspecified
M6200	Separation of muscle (nontraumatic) unspecified site
M726	Necrotizing fasciitis
M797	Fibromyalgia
M7989	Other specified soft tissue disorders
M810	Age-related osteoporosis without current pathological fracture
N131	Hydronephrosis with ureteral stricture not elsewhere classified
N1330	Unspecified hydronephrosis
N170	Acute kidney failure with tubular necrosis
N179	Acute kidney failure unspecified
N183	Chronic kidney disease stage 3 (moderate)
N186	End stage renal disease
N2581	Secondary hyperparathyroidism of renal origin
N281	Cyst of kidney acquired
N289	Disorder of kidney and ureter unspecified
N3001	Acute cystitis with hematuria
N390	Urinary tract infection site not specified
N401	Benign prostatic hyperplasia with lower urinary tract symptoms
N809	Endometriosis unspecified
N854	Malposition of uterus
N9410	Unspecified dyspareunia
P271	Bronchopulmonary dysplasia originating in the perinatal period
P2889	Other specified respiratory conditions of newborn
P612	Anemia of prematurity
Q828	Other specified congenital malformations of skin
Q8500	Neurofibromatosis unspecified
R000	Tachycardia unspecified
R001	Bradycardia unspecified
R042	Hemoptysis
R0689	Other abnormalities of breathing
R0789	Other chest pain
R079	Chest pain unspecified
R0902	Hypoxemia
R112	Nausea with vomiting unspecified
R1310	Dysphagia unspecified
R17	Unspecified jaundice
R180	Malignant ascites
R188	Other ascites
R310	Gross hematuria
R319	Hematuria unspecified
R338	Other retention of urine
R339	Retention of urine unspecified
R34	Anuria and oliguria
R410	Disorientation unspecified
R4182	Altered mental status unspecified
R52	Pain unspecified
R55	Syncope and collapse
R600	Localized edema
R627	Adult failure to thrive
R633	Feeding difficulties
R636	Underweight
R6510	Systemic inflammatory response syndrome (SIRS) of non-infectious origin without acute organ dysfunction
R6520	Severe sepsis without septic shock
R6521	Severe sepsis with septic shock
R739	Hyperglycemia unspecified
R740	Nonspecific elevation of levels of transaminase and lactic acid dehydrogenase [LDH]
R748	Abnormal levels of other serum enzymes
R7881	Bacteremia
R791	Abnormal coagulation profile
S301XXA	Contusion of abdominal wall initial encounter
S31105A	Unspecified open wound of abdominal wall periumbilic region without penetration into peritoneal cavity initial encounter
T380X5A	Adverse effect of glucocorticoids and synthetic analogues initial encounter
T402X5A	Adverse effect of other opioids initial encounter
T45515A	Adverse effect of anticoagulants initial encounter
T50995A	Adverse effect of other drugs medicaments and biological substances initial encounter
T8132XA	Disruption of internal operation (surgical) wound not elsewhere classified initial encounter
T814XXA	Infection following a procedure initial encounter
T8182XA	Emphysema (subcutaneous) resulting from a procedure initial encounter
T8579XA	Infection and inflammatory reaction due to other internal prosthetic devices implants and grafts initial encounter
T8612	Kidney transplant failure
X58XXXA	Exposure to other specified factors initial encounter
Y813	Surgical instruments materials and general- and plastic-surgery devices (including sutures) associated with adverse incidents
Y832	Surgical operation with anastomosis bypass or graft as the cause of abnormal reaction of the patient or of later complication without mention of misadventure at the time of the procedure
Y838	Other surgical procedures as the cause of abnormal reaction of the patient or of later complication without mention of misadventure at the time of the procedure
Y848	Other medical procedures as the cause of abnormal reaction of the patient or of later complication without mention of misadventure at the time of the procedure
Y92239	Unspecified place in hospital as the place of occurrence of the external cause
Z23	Encounter for immunization
Z412	Encounter for routine and ritual male circumcision
Z590	Homelessness
Z66	Do not resuscitate
Z681	Body mass index (BMI) 19.9 or less adult
Z6825	Body mass index (BMI) 25.0-25.9 adult
Z6830	Body mass index (BMI) 30.0-30.9 adult
Z6831	Body mass index (BMI) 31.0-31.9 adult
Z6832	Body mass index (BMI) 32.0-32.9 adult
Z6838	Body mass index (BMI) 38.0-38.9 adult
Z6839	Body mass index (BMI) 39.0-39.9 adult
Z6841	Body mass index (BMI) 40.0-44.9 adult
Z6842	Body mass index (BMI) 45.0-49.9 adult
Z6843	Body mass index (BMI) 50-59.9 adult
Z6844	Body mass index (BMI) 60.0-69.9 adult
Z716	Tobacco abuse counseling
Z7401	Bed confinement status
Z751	Person awaiting admission to adequate facility elsewhere
Z781	Physical restraint status
Z7901	Long term (current) use of anticoagulants
Z7902	Long term (current) use of antithrombotics/antiplatelets
Z794	Long term (current) use of insulin
Z7951	Long term (current) use of inhaled steroids
Z7952	Long term (current) use of systemic steroids
Z79891	Long term (current) use of opiate analgesic
Z800	Family history of malignant neoplasm of digestive organs
Z809	Family history of malignant neoplasm unspecified
Z830	Family history of human immunodeficiency virus [HIV] disease
Z8349	Family history of other endocrine nutritional and metabolic diseases
Z85038	Personal history of other malignant neoplasm of large intestine
Z85048	Personal history of other malignant neoplasm of rectum rectosigmoid junction and anus
Z85528	Personal history of other malignant neoplasm of kidney
Z85828	Personal history of other malignant neoplasm of skin
Z85850	Personal history of malignant neoplasm of thyroid
Z8614	Personal history of Methicillin resistant Staphylococcus aureus infection
Z8619	Personal history of other infectious and parasitic diseases
Z8669	Personal history of other diseases of the nervous system and sense organs
Z86711	Personal history of pulmonary embolism
Z86718	Personal history of other venous thrombosis and embolism
Z8673	Personal history of transient ischemic attack (TIA) and cerebral infarction without residual deficits
Z8739	Personal history of other diseases of the musculoskeletal system and connective tissue
Z87898	Personal history of other specified conditions
Z881	Allergy status to other antibiotic agents status
Z882	Allergy status to sulfonamides status
Z885	Allergy status to narcotic agent status
Z886	Allergy status to analgesic agent status
Z888	Allergy status to other drugs medicaments and biological substances status
Z902	Acquired absence of lung [part of]
Z90410	Acquired total absence of pancreas
Z9049	Acquired absence of other specified parts of digestive tract
Z905	Acquired absence of kidney
Z90710	Acquired absence of both cervix and uterus
Z9081	Acquired absence of spleen
Z91040	Latex allergy status
Z9114	Patients other noncompliance with medication regimen
Z9119	Patients noncompliance with other medical treatment and regimen
Z915	Personal history of self-harm
Z9181	History of falling
Z923	Personal history of irradiation
Z932	Ileostomy status
Z940	Kidney transplant status
Z950	Presence of cardiac pacemaker
Z951	Presence of aortocoronary bypass graft
Z953	Presence of xenogenic heart valve
Z955	Presence of coronary angioplasty implant and graft
Z95810	Presence of automatic (implantable) cardiac defibrillator
Z95828	Presence of other vascular implants and grafts
Z9689	Presence of other specified functional implants
Z982	Presence of cerebrospinal fluid drainage device
Z9851	Tubal ligation status
Z9884	Bariatric surgery status
Z992	Dependence on renal dialysis
Z993	Dependence on wheelchair
Z9981	Dependence on supplemental oxygen
ICD-10-CM procedural codes	
0WUF0JZ	Supplement Abdominal Wall with Synth Sub Open Approach
0WUF4JZ	Supplement Abdominal Wall with Synth Sub Perc Endo Approach
30233N1	Transfuse Nonaut Red Blood Cells in Periph Vein Perc
0DN80ZZ	Release Small Intestine Open Approach
0DNW0ZZ	Release Peritoneum Open Approach
02HV33Z	Insertion of Infusion Dev into Sup Vena Cava Perc Approach
0WQF0ZZ	Repair Abdominal Wall Open Approach
0DB80ZZ	Excision of Small Intestine Open Approach
0YU50JZ	Supplement R Inguinal Region with Synth Sub Open Approach
0WPF0JZ	Removal of Synthetic Substitute from Abd Wall Open Approach
0KNK0ZZ	Release Right Abdomen Muscle Open Approach
0KNL0ZZ	Release Left Abdomen Muscle Open Approach
0BH17EZ	Insertion of Endotracheal Airway into Trachea Via Opening
0YU60JZ	Supplement L Inguinal Region with Synth Sub Open Approach
0DNS0ZZ	Release Greater Omentum Open Approach
0BUS4JZ	Supplement Left Diaphragm with Synthetic Substitute Percutan
8E0W4CZ	Robotic Assisted Procedure of Trunk Perc Endo Approach
0HB7XZZ	Excision of Abdomen Skin External Approach
0BUR4JZ	Supplement Right Diaphragm with Synthetic Substitute Percuta
0YQ50ZZ	Repair Right Inguinal Region Open Approach
0DBS0ZZ	Excision of Greater Omentum Open Approach
0JN80ZZ	Release Abd Subcu/Fascia Open Approach
0WBF0ZZ	Excision of Abdominal Wall Open Approach
0WUF0KZ	Supplement Abdominal Wall with Nonaut Sub Open Approach
0YQA0ZZ	Repair Bilateral Inguinal Region Open Approach
3E0436Z	Introduction of Nutritional into Central Vein Perc Approach
0WUF07Z	Supplement Abdominal Wall with Autol Sub Open Approach
0DQ80ZZ	Repair Small Intestine Open Approach
0DNW4ZZ	Release Peritoneum Percutaneous Endoscopic Approach
2W13X6Z	Compression of Abdominal Wall using Pressure Dressing
5A1955Z	Respiratory Ventilation Greater than 96 Consecutive Hours
5A1D60Z	Performance of Urinary Filtration Multiple
0WQF4ZZ	Repair Abdominal Wall Percutaneous Endoscopic Approach
0DNB0ZZ	Release Ileum Open Approach
5A1945Z	Respiratory Ventilation 24-96 Consecutive Hours
0DN84ZZ	Release Small Intestine Percutaneous Endoscopic Approach
0D9670Z	Drainage of Stomach with Drainage Device Via Opening
0YQ60ZZ	Repair Left Inguinal Region Open Approach
30233K1	Transfuse Nonaut Frozen Plasma in Periph Vein Perc
0JB80ZZ	Excision of Abd Subcu/Fascia Open Approach
0DNE0ZZ	Release Large Intestine Open Approach
0DNS4ZZ	Release Greater Omentum Percutaneous Endoscopic Approach
0YU54JZ	Supplement R Inguinal Region w Synth Sub Perc Endo
0KXL0Z6	Transfer Left Abdomen Muscle TRAM Flap Open Approach
0BJ08ZZ	Inspection of Tracheobronchial Tree Endo
0DH63UZ	Insertion of Feeding Device into Stomach Perc Approach
3E0G76Z	Introduction of Nutritional into Up GI Via Opening
3E0336Z	Introduction of Nutritional into Periph Vein Perc Approach
0KXK0Z6	Transfer Right Abdomen Muscle TRAM Flap Open Approach
8E0ZXY6	Isolation
3E03329	Introduce of Oth Anti-infect into Periph Vein Perc Approach
0DTJ0ZZ	Resection of Appendix Open Approach
0W9G3ZZ	Drainage of Peritoneal Cavity Percutaneous Approach
0FT40ZZ	Resection of Gallbladder Open Approach
4A133B1	Monitoring of Arterial Pressure Peripheral Perc Approach
0JX80ZZ	Transfer Abd Subcu/Fascia Open Approach
0BUS0JZ	Supplement Left Diaphragm with Synthetic Substitute Open App
3E0234Z	Introduction of Serum/Tox/Vaccine into Muscle Perc Approach
0VTTXZZ	Resection of Prepuce External Approach
B548ZZA	Ultrasonography of Superior Vena Cava Guidance
3E1M38Z	Irrigation of Peritoneal Cavity using Irrigat Perc Approach
BW21YZZ	CT Scan of Abd & Pelvis using Oth Contrast
0YQA4ZZ	Repair Bilateral Inguinal Region Perc Endo Approach
0YUA4JZ	Supplement Bi Inguinal Region w Synth Sub Perc Endo
0KNL4ZZ	Release Left Abdomen Muscle Perc Endo Approach
0WJG4ZZ	Inspection of Peritoneal Cavity Perc Endo Approach
0JR807Z	Replace of Abd Subcu/Fascia with Autol Sub Open Approach
0KXL0Z2	Transfer Left Abdomen Muscle with Skin Subcu Open Approach
0DBL0ZZ	Excision of Transverse Colon Open Approach
0JQ80ZZ	Repair Abdomen Subcutaneous Tissue and Fascia Open Approach
0DH64UZ	Insertion of Feeding Device into Stomach Perc Endo Approach
3E0F7GC	Introduce of Oth Therap Subst into Resp Tract Via Opening
0DBN0ZZ	Excision of Sigmoid Colon Open Approach
0DJD8ZZ	Inspection of Lower Intestinal Tract Endo
30233R1	Transfuse Nonaut Platelets in Periph Vein Perc
4A033R1	Measure of Arterial Saturation Peripheral Perc Approach
0BUR0JZ	Supplement Right Diaphragm with Synthetic Substitute Open Ap
0DBB0ZZ	Excision of Ileum Open Approach
0DN60ZZ	Release Stomach Open Approach
0KNK4ZZ	Release Right Abdomen Muscle Perc Endo Approach
0DNL0ZZ	Release Transverse Colon Open Approach
5A2204Z	Restoration of Cardiac Rhythm Single
3E033XZ	Introduction of Vasopressor into Periph Vein Perc Approach
4A1GXSH	Monitor Skin/Breast Vasc Perfus ICG dye Extern
0FN00ZZ	Release Liver Open Approach
30233L1	Transfuse Nonaut Fresh Plasma in Periph Vein Perc
5A1D00Z	Performance of Urinary Filtration Single
0DB84ZZ	Excision of Small Intestine Perc Endo Approach
0DNT0ZZ	Release Lesser Omentum Open Approach
3E0R3BZ	Introduction of Anesthetic into Spinal Canal Perc Approach
5A09457	Assistance with Respiratory Ventilation 24-96 Hrs CPAP
0WJG0ZZ	Inspection of Peritoneal Cavity Open Approach
05H633Z	Insertion of Infusion Dev into L Subclav Vein Perc Approach
0DBB4ZZ	Excision of Ileum Percutaneous Endoscopic Approach
30233M1	Transfuse Nonaut Plasma Cryoprecip in Periph Vein Perc
0TQB0ZZ	Repair Bladder Open Approach
08J0XZZ	Inspection of Right Eye External Approach
08J1XZZ	Inspection of Left Eye External Approach
0KUL07Z	Supplement Left Abdomen Muscle with Autol Sub Open Approach
0DNN0ZZ	Release Sigmoid Colon Open Approach
0DV40ZZ	Restriction of Esophagogastric Junction Open Approach
0KXK0Z2	Transfer R Abd Muscle with Skin Subcu Open Approach
0DB80ZX	Excision of Small Intestine Open Approach Diagnostic
0DBT0ZZ	Excision of Lesser Omentum Open Approach
0DQE0ZZ	Repair Large Intestine Open Approach
0DTF0ZZ	Resection of Right Large Intestine Open Approach
0KUK07Z	Supplement R Abd Muscle with Autol Sub Open Approach
05HN33Z	Insert Infusion Dev in L Int Jugular Vein Perc
06HM33Z	Insertion of Infusion Dev into R Femor Vein Perc Approach
0DCW0ZZ	Extirpation of Matter from Peritoneum Open Approach
0DH60UZ	Insertion of Feeding Device into Stomach Open Approach
0DS60ZZ	Reposition Stomach Open Approach
0W9G0ZZ	Drainage of Peritoneal Cavity Open Approach
0YQ84ZZ	Repair Left Femoral Region Percutaneous Endoscopic Approach
03HB33Z	Insertion of Infusion Dev into R Radial Art Perc Approach
0CJS8ZZ	Inspection of Larynx Endo
0FB03ZX	Excision of Liver Percutaneous Approach Diagnostic
0J9800Z	Drainage of Abd Subcu/Fascia with Drain Dev Open Approach
0W9G3ZX	Drainage of Peritoneal Cavity Percutaneous Approach Diagn
30233P1	Transfuse Nonaut Frozen Red Cells in Periph Vein Perc
HZ2ZZZZ	Detoxification Services for Substance Abuse Treatment
CPT (Current Procedural Terminology®) codes	
J3010	Fentanyl citrate injeciton
J2405	Ondansetron hcl injection
J0690	Cefazolin sodium injection
J1100	Dexamethasone sodium phos
J1885	Ketorolac tromethamine inj
49505	Prp i/hern init reduc >5 yr
J7120	Ringers lactate infusion
80053	Comprehen metabolic panel
83735	Assay of magnesium
G0378	Hospital observation per hr
94640	Airway inhalation treatment
99281	Emergency dept visit
84100	Assay of phosphorus
85730	Thromboplastin time partial
J1200	Diphenhydramine hcl injectio
83690	Assay of lipase
81001	Urinalysis auto w/scope
47562	Laparoscopic cholecystectomy
C9290	Injection, bupivacaine liposome, 1 mg
49565	Rerepair ventrl hern reduce
74177	Ct abd & pelv w/contrast
99285	Emergency dept visit
83605	Assay of lactic acid
71010	Chest x-ray 1 view frontal
84484	Assay of troponin quant
99284	Emergency dept visit
J7040	Normal saline solution infus
49520	Rerepair ing hernia reduce
J3480	Inj potassium chloride
J2060	Lorazepam injection
G0237	Therapeutic procd strg endur
J2543	Piperacillin/tazobactam
49329	Laparo proc abdm/per/oment
80076	Hepatic function panel
94660	Pos airway pressure cpap
74176	Ct abd & pelvis w/o contrast
82150	Assay of amylase
94664	Evaluate pt use of inhaler
J1580	Garamycin gentamicin inj
44970	Laparoscopy appendectomy
J1956	Levofloxacin injection
82550	Assay of ck (cpk)
84702	Chorionic gonadotropin test
49500	Rpr ing hernia init reduce
J3360	Diazepam injection
82553	Creatine mb fraction
80301	Presumptive Drug Class Screening Procedures
J1630	Haloperidol injection
Q0163	Diphenhydramine hcl 50mg
47563	Laparo cholecystectomy/graph
99283	Emergency dept visit
J7512	Prednisone ir or dr oral 1mg
71275	Ct angiography chest
J3411	Thiamine hcl 100 mg
93306	Tte w/doppler complete
74022	X-ray exam series abdomen
76000	Fluoroscope examination
93975	Vascular study
97003	occupational therapy evaluation
86920	Compatibility test spin
11005	Debride abdom wall
51702	Insert temp bladder cath
J2597	Inj desmopressin acetate
86960	Vol reduction of blood/prod
11008	Remove mesh from abd wall
A4649	Surgical supplies
S0028	Injection, famotidine, 20 mg
J1741	Ibuprofen injection
49083	Abd paracentesis w/imaging
72170	X-ray exam of pelvis
S0074	Injection, cefotetan disodiu
52281	Cystoscopy and treatment
82140	Assay of ammonia
87106	Fungi identification yeast
J2785	Regadenoson injection
